# Changes in Oxidative Stress Markers and Biological Markers of Muscle Injury with Aging at Rest and in Response to an Exhaustive Exercise

**DOI:** 10.1371/journal.pone.0090420

**Published:** 2014-03-11

**Authors:** Mohamed Amine Bouzid, Omar Hammouda, Regis Matran, Sophie Robin, Claudine Fabre

**Affiliations:** 1 Université Lille Nord de France, EA 4488, Activité Physique Muscle Santé, Faculté des Sciences du Sport et de l'Education Physique, Ronchin, France; 2 Research Laboratory «Sport Performance Optimisation», National Center of Medecine and Science in Sports, Tunis, Tunisia; 3 Service EFR, CHRU de Lille, Lille, France; Temple University, United States of America

## Abstract

**Purpose:**

The aim of this study was to evaluate whether oxidative stress markers and biomarkers of muscle injury would be affected by aging at rest and in response to an incremental exhaustive exercise.

**Methods:**

Fifteen young (20.3±2.8 years) and fifteen older adults (65.1±3.5 years) performed an incremental cycle ergometer test to exhaustion. Before and after exercise, oxidative stress [superoxide dismutase (SOD), glutathione peroxidase (GPX), glutathione reductase (GR), ascorbic acid, α-Tocopherol, malondialdehyde (MDA)] and muscle injury [creatine kinase (CK), lactate deshydrogenase (LDH)] biomarkers were assessed.

**Results:**

At rest, there was no difference in oxidative stress markers and LDH level between the groups, however CK was significantly higher in the young group than the elderly group (p<0.05). During recovery, in comparison with resting values, a significant increase in SOD (1092±145.9 vs. 1243±98 U/g Hb), GPX (67.4±12.7 vs. 79.2±15.6 U/g Hb) and GR (6.5±0.9 vs. 7.7±0.5 U/g Hb) activities were observed only in the young group (p<0.05). MDA has increased only in the older group (0.54±0.2 vs. 0.79±0.2 µmol/l) (p<0.01). CK increased in both groups (young group: 122.5±22.2 vs. 161.9±18.7 UI/l; older group: 88.8±34.1 vs. 111.1±25.9 UI/l) (p<0.01), however LDH has increased only in the young group (400.5±22.2 vs. 485±18.7 UI/l) (p<0.01) without alteration in the older group (382.8±34.1 vs. 418.5±25.9 UI/l).

**Conclusions:**

These findings indicate that aging is associated with a decrease in antioxidant efficiency and an increase in oxidative stress damage. Furthermore, older adults would not more susceptible to exercise-induced muscle injury than young people.

## Introduction

Aging has been hypothesized to be partially caused by the deleterious and cumulative effects of reactive oxygen species (ROS) occurring throughout the life span [1]. Numerous studies have shown that elderly people are under constant and increasing assault by ROS, as indicated by enhanced lipid peroxidation, protein oxidation, and alteration of antioxidant enzyme activities [2], [3], [4]. These studies have used comparisons between older and young subjects to evaluate aging effects on oxidative stress [5], [6]. However, most of them have investigated oxidative stress markers in resting conditions and results emerging from these studies are always contradictory.

Moreover, advanced age lead to a decrease in muscle mass and strength, a process called sarcopenia [7]. Evidence from animal studies has reported that an accumulation of oxidative stress damage in mitochondria, proteins and DNA during aging is a potential cause of sarcopenia and muscle damage [8]. In this context, several studies have investigated the effects of ageing on potential muscle damage in humans and showed conflicting results [9], [10].

On the other hand, acute exercise has the capacity to increase the generation of ROS and to induce muscle damage especially for single bouts of exhaustive exercise [11]. These results could be more pronounced for the elderly population where sedentary life style and age-related physiological dysfunctions could impair antioxidant defense and increase susceptibility to oxidative stress and muscle damage. However, it seems to be only few studies on human who investigated exercise-induced oxidative stress and muscle damage in elderly subjects. In this sense, Di Massimo et al [12] and Toft et al [13] reported an increase in oxidative stress (thiobarbituric acid–reactive substances) and muscle injury (i.e., creatine kinase (CK) and myoglobin) markers in response to acute exercise in elderly subjects. Similarly, it has been demonstrated that acute running treadmill exercise increases oxidative stress (i.e., malondialdehyde (MDA) and F2-isoprostanes) and muscle injury markers (CK) in healthy older adults [10]. In this context, a positive correlation between markers of muscle damage and free-radical production has been previously shown [10], supporting the hypothesis that free radicals produced during exercise alter muscle cell membrane permeability [14]. Additionally, a positive correlation between increasing thiobarbituric acid–reactive substances and creatine kinase was found in an earlier animal study during exercise [15] indicating that biomarkers of muscle damage are indicative of oxidative stress [16].

Given that aging and acute exercise exhibit prooxidant potential and stronger factor of muscle damage, it would therefore seem reasonable to expect a more pronounced exercise-induced oxidative stress response and biomarkers of muscle damage in elderly compared with young subjects. Results from the few studies that have tested this hypothesis remained unclear and were not performed in humans. Ji et al [17] have demonstrated a similar response of oxidative stress markers to running exercise in young and old rats. However, the study of Bejma et al [18] showed a higher response of oxidative stress markers in old rats compared with younger ones after running exercise. In order to provide an answer to this question, we aimed to investigate both resting and exercise-induced oxidative stress markers and biomarkers of muscle damage in a sample of healthy older adults and young subjects. We hypothesized that young subjects would have a better antioxidant activity and lower levels of biomarkers indicative of muscle damage at rest and following an incremental exhaustive exercise, than the older adults.

## Materials and Methods

### Participants

Fifteen young adults (20.3±2.8 yr; 9 males and 6 females) and fifteen older adults (65.1±3.5 yr; 7 males and 8 females) (Table 1) were included in the study. Both groups were matched for body weight, height, and dietary status and were classified as sedentary since they had not regularly engaged in any physical activities over the past three years.

**Table 1:** Anthropometric and physiological parameters for the two groups (mean ± SD).

	Young group	Elderly group
**Age (years)**	20.3±2.8	65.1±3.5##
**Weight (Kg)**	66.1±11.7	71.8±7.6
**Height (m)**	1.7±0.2	1.6±0.1
**Fat (%)**	25.8±2.7	28.4±4.2
**VO_2max_ (ml/min/kg)**	44.2±5.2	23.2±4.4##
**Pmax (Watts)**	220.7±36.4	94.1±32.6##

## : significant difference between young and elderly subjects at p<0.01.

### Ethical Considerations

After receiving a description of the protocol, and its benefits and possible risks, each volunteer (and parents/tutors for the minors) provided a written informed consent. The study was conducted according to the Declaration of Helsinki and the protocol was fully approved by the Ethics Committee of the Regional University Hospital Centre (CHRU) of Lille before the commencement of the assessments (Permit number: N° 07/42).

### Protocol

On the arrival of the subjects to the hospital, a clinical interview, an electrocardiogram (ECG) at rest, a skin fold to measure the percentage of fat mass and a record of blood pressure were carried out to verify the absence of exclusion criteria. These criteria were: inflammatory disorders, recent infections, renal or hepatic insufficiency, active coronary artery disease, diabetes, heart failure, hormonal replacement therapy, or supplementation with antioxidants within 3 months before the study. In the other hand, to be included to the study, the age range for the young participants was 17–30 years. For the elderly group, participants needed to be at least 60 years old to participate in this study.

After that, a nurse placed a venous catheter in the forearm of the subject to take a blood sample at rest. Finally, participants performed an incremental exercise test followed by a period of recovery, when blood samples were once again collected at 5 and at 20 minutes after the end of exercise. The parameters measured were: oxidative stress markers: superoxide dismutase (SOD), glutathione peroxidase (GPX), glutathione reductase (GR), ascorbic acid, α-Tocopherol and MDA and biomarkers of muscle injury: CK and lactate deshydrogenase (LDH).

### Exercise testing

Each subject performed an incremental exercise test until exhaustion on an electrically braked cycle ergometer (Excalibur Sport, Lode B.V, Medical Technology, Groningen, Netherlands) connected to a computer software (Ergocard, Medisoft, Dinant, Belgium). After resting for 3-minutes on the cycle ergometer, the participant started the exercise at 30 watts (W) and 60–70 revolutions per minute of pedaling for 3 minutes as a warm-up period. Next, for the elderly group, the exercise was performed with a workload increase every 3 minutes of 20 W for men and 10 W for women. For the young group, the workload was increased every 3 minutes by 30 W for men and women until the end of exercise testing. For the two groups, the incremental exercise was followed by a 2-minute active recovery at 25 W and a 3-minute passive recovery period. This workload increase was chosen in order to insure equality in exercise testing duration between the groups. This procedure, designed to reach maximal oxygen uptake (VO_2max_) in young and older adults, was selected in previous studies [19], [20] and is the most likely task that induces oxidative stress [21]. For each subject, the task duration was calculated without including the warm-up and recovery periods.

The incremental test was considered as maximum if at least three of the following criteria were observed: 1) exhaustion of the subject or inability to maintain the required pedaling speed in spite verbal encouragement, 2) (VO_2max_) plateau was reached and 3) the predicted maximum heart rate (HR max) (208−0.7×age ±10% [22]) was achieved.

Gas exchanges (oxygen consumption: VO_2_, carbon dioxide production: VCO_2_, and ventilation: VE) were measured continuously throughout the test and for 5 minutes during recovery, using a gas-exchange system analyzer (Medisoft, Belgium). Blood pressure was measured at rest and every 3 minutes during exercise and recovery.

### Diet record

A 4-day food record was completed by each subject on a notebook. At the start, a standardized individual information session was organized to give subjects instructions on how to record their daily food intake. Food quantities were estimated, specifying the number of units and a code corresponding to the size of the portion, using a reference portion guideline book. The average nutrient diet content was calculated using Nutrilog Micro software (Version 2.32, France). Intakes of vitamin C and vitamin E were estimated with the use of the French database of ANSES Ciqual (France).

### Blood sampling

In order to avoid time-of-day effects on oxidative stress and muscle damage markers, as demonstrated in previous research [23], all exercise sessions and blood samples were performed at the same time of day (14:00 pm). A blood sample was drawn at rest and 5 minutes following the exercise for the measurements of MDA, LDH and CK levels and at 20 minutes after exercise to measure SOD, GPX, GR, ascorbic acid and α-Tocopherol levels. Blood samples drawn 20 minutes after the exercise was chosen in view of research studies demonstrating that immediately after the exercise, no change on antioxidant capacity was observed [24]. However, the main change of antioxidant capacity has been observed from 20 minutes post exercise [25]. Blood samples were drawn from the antecubital vein into a dry tube to measure α-Tocopherol, into an EDTA tube for ascorbic acid, MDA, CK and LDH and into a heparinized tube for SOD, GPX and GR. Plasma was obtained by centrifugation of blood at 3000 rpm for 10 minutes at 4°C. α-Tocopherol, ascorbic acid, CK, LDH and MDA levels were evaluated in plasma. However, SOD, GPX and GR activities were evaluated in blood erythrocyte.

#### SOD, GPX and GR

SOD activity was analyzed using a clinical chemical analyzer (Konelab 60, Thermo Fisher Scientific Society). This method uses xanthine and xanthine oxidase to generate superoxyde radicals that react with 2-(4-iodophenyl-)-3-(4-nitrophenyl)-5-phenyltetrazoluim chloride to form a red formazan dye. The SOD activity is then quantified by measuring the degree of inhibition of this reaction.

GPX activity was measured using a reagent set (RANSEL, society RANDOX). GPX activity is measured indirectly by a coupled reaction with GR. Oxidized glutathione (GSSG), produced upon reduction of an organic hydroperoxide by GPX, is recycled to its reduced state by GR and nicotinamide adenine dinucleotide phosphate (NADPH). The oxidation of NADPH to NADP^+^ is accompanied by a decrease in absorbance at 340 nm. The rate of decrease is directly proportional to the GPX activity in the sample.

GR activity was measured using a clinical chemical analyzer (Konelab 60). GR is a flavoprotein that catalyzes the reaction, whereby reduced NADPH converts GSSG to reduced glutathione (GSH). We evaluated this enzymatic activity with a GR determining the rate of NADPH oxidation. The oxidation of NADPH to NADP^+^ results in a decreased absorbance at 340 nm that is directly proportional to the GR activity.

#### α-Tocopherol

α-Tocopherol was measured in the plasma by the high performance liquid chromatography technique with spectrophotometric detection. In this technique, α-Tocopherol is extracted with heptanes after protein precipitation by ethanol and then injected into HPLC system. The selected wavelength was 292 nm.

#### Ascorbic acid

After deproteinization of plasma with metaphosphoric acid, ascorbic acid was oxidized to ascorbic dehydroacide by ascorbate oxidase (DHAA).The DHAA bound to the O-phenylene-diamine (OPDA), which produced a chromophore whose absorbance has read at 340 nm on a clinical chemical analyzer (Konelab 60). Thus, the rate of ascorbic acid in plasma was deduced from the amount of chromophore formed.

#### Malondialdehyde

The MDA was measured in plasma by the technique of HPLC with fluorimetric detection. It was determined by modified thiobarbituric acid (TBARS). 100 µl of plasma was precipitated with trichloroacetic acid (200 µl) and mixed with 450 µl of normal saline solution (0.9%). The whole mixture was incubated in a 90°C water bath for 30 min, then cooled in water. After centrifugation at 3000 rpm for 5 min at 4°C, the absorbance was read at 532 nm.

#### CK and LDH

CK activity was determined spectrophotometrically by measuring NADPH formed by hexokinase and the glucose- 6-phosphate dehydrogenase coupled enzymatic system. LDH activity was determined by measuring NADH consumption using the reagent kits.

### Statistical methods

After confirmation of distribution normality using Shapiro-Wilk tests, data were analyzed by a two-way repeated measures analysis of variance: “groups” (young-elderly) versus “time” (rest-recovery) factors. When the ANOVA F ratio was significant, the means were compared using pair-wise multiple comparison procedures (Bonferroni post-hock test). To compare cardiorespiratory adaptations to the exercise, anthropometric values and dietary intakes data between groups, a T-test for independent samples was performed. Pearson correlation test was used to examine correlation between oxidative stress markers and biomarkers of muscle injury. Significance was established at p<0.05 and data are presented as means ± SD.

## Results

### Characteristics of the subjects and performance during exhaustive exercise

Young subjects demonstrated a significant higher value in maximal oxygen uptake (VO_2max_) and maximal aerobic power (Pmax) in comparison with the older group (p<0.01) (Table 1).

No significant difference was observed in the duration of the exercise testing between young and older groups: 19.2±3.6 min and 16.8±5.4 min, respectively.

### Dietary antioxidant micronutrient intakes

Statistical analysis showed no significant difference in vitamin C and vitamin E intake between the studied groups. Vitamin C intake was 90±34 mg/day for the young group and 101±27 mg/day for the older group. Vitamin E intake was 7.8±2.2 mg/day for the young group and 8.5±3.6 mg/day for the older group.

### Biologicals markers

**Figure 1:**
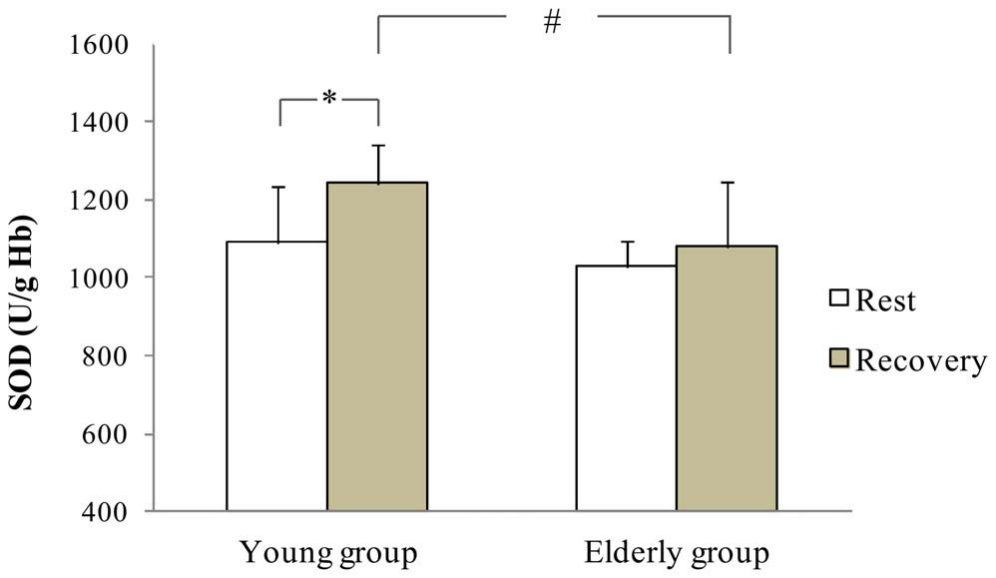
SOD at rest and at the recovery in the young and the elderly subjects. *: significant difference between rest and recovery period (p<0.05). #: significant difference between young and elderly subjects (p<0.05).

**Figure 2:**
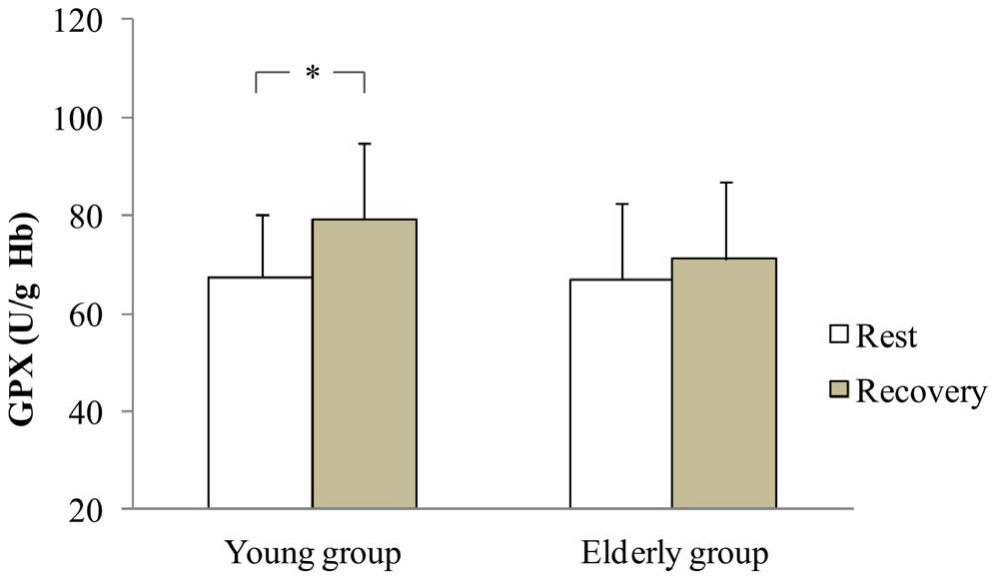
GPX at rest and at the recovery in the young and the elderly subjects. *: significant difference between rest and recovery period (p<0.05).

At rest, statistical analysis showed no difference in antioxidant enzymes activities between groups. During recovery, antioxidant enzymes activities increased only in the young group: SOD (+13.8%), GPX (+17.5%) and GR (+18.4%) (p<0.05 Fig. 1, 2, 3 respectively). In addition, the young group presented higher SOD and GR values than the older group during recovery (p<0.05). Regarding non enzymatic antioxidants, statistical analysis showed no exercise or aging effects on ascorbic acid and α-Tocopherol levels (Fig. 4).

**Figure 3:**
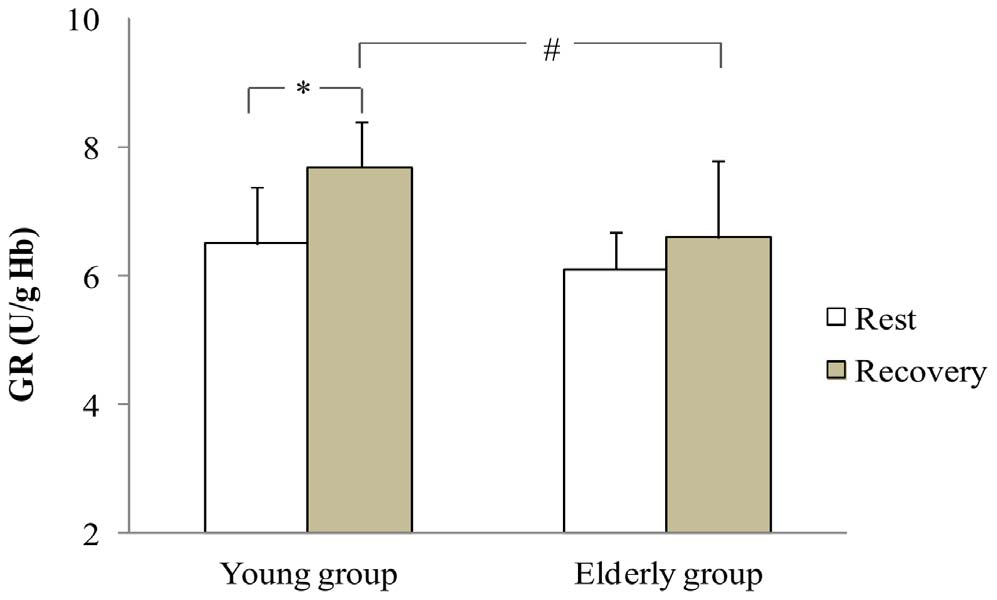
GR at rest and at the recovery in the young and the elderly subjects. *: significant difference between rest and recovery period (p<0.05). #: significant difference between young and elderly subjects (p<0.05).

**Figure 4:**
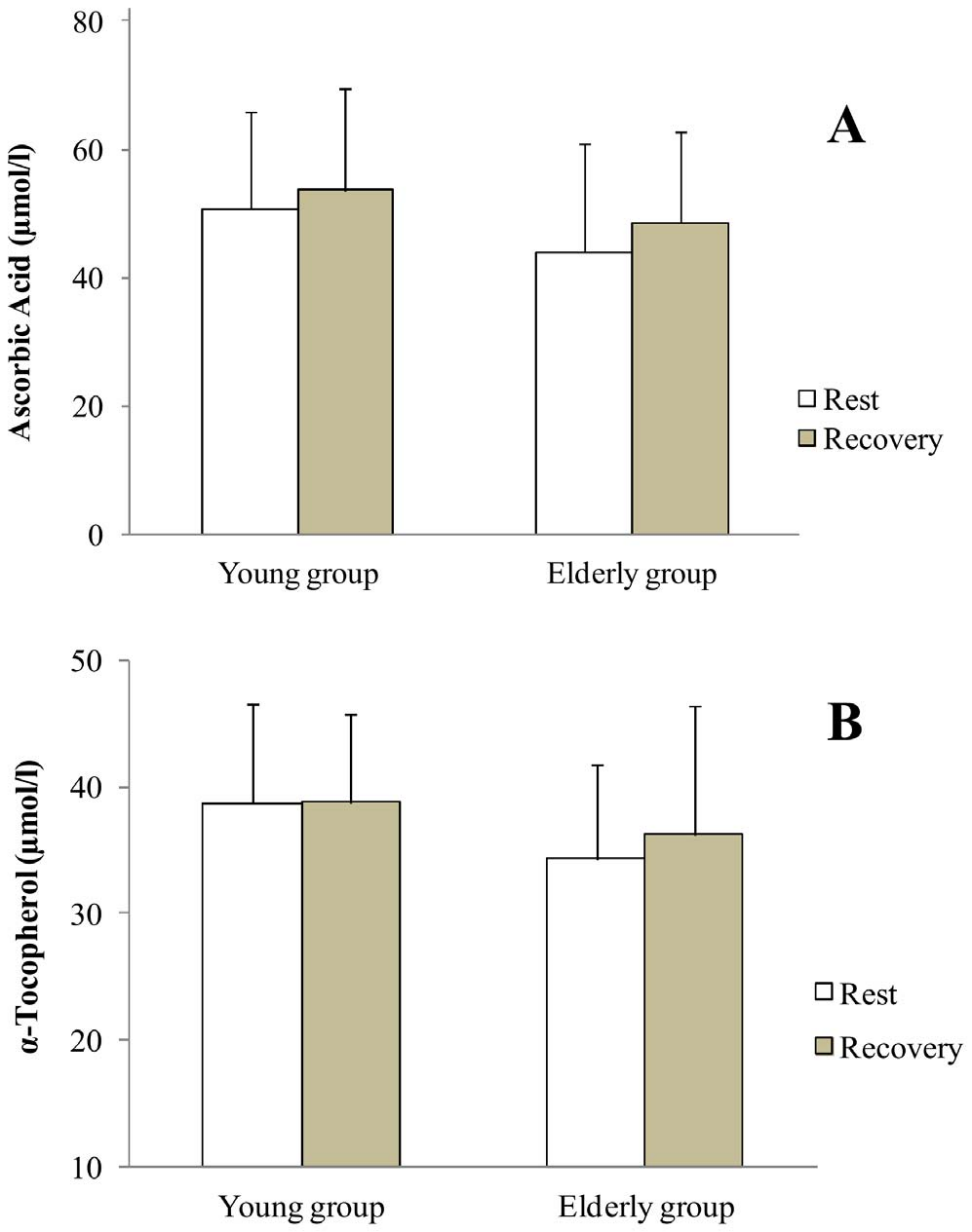
Ascorbic acid and α-Tocopherol at rest and at the recovery in the young and the elderly subjects. A = Ascorbic acid, B = α-Tocopherol.

**Figure 5:**
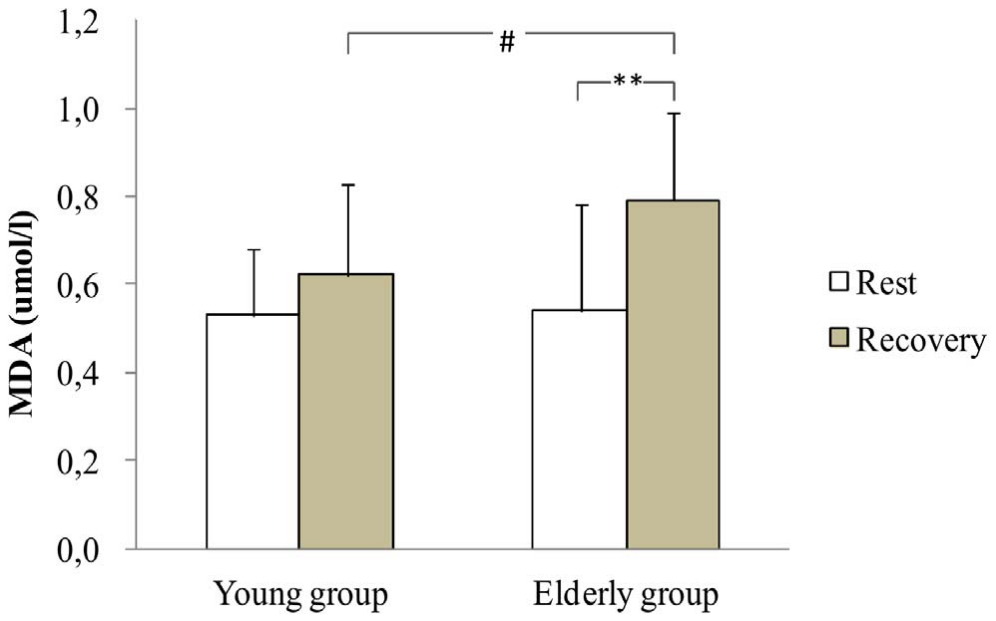
MDA at rest and at the recovery in the young and the elderly subjects. **: significant difference between rest and recovery period (p<0.01). #: Significant difference between young and elderly subjects (p<0.05).

Concerning MDA, resting levels presented no significant difference in both groups. During recovery, MDA levels increased by 46.2% in the old group (p<0.01 Fig. 5) without change in the young group. In addition, the older group presented a higher MDA values than the young group during recovery (p<0.05 Fig. 5)

**Figure 6:**
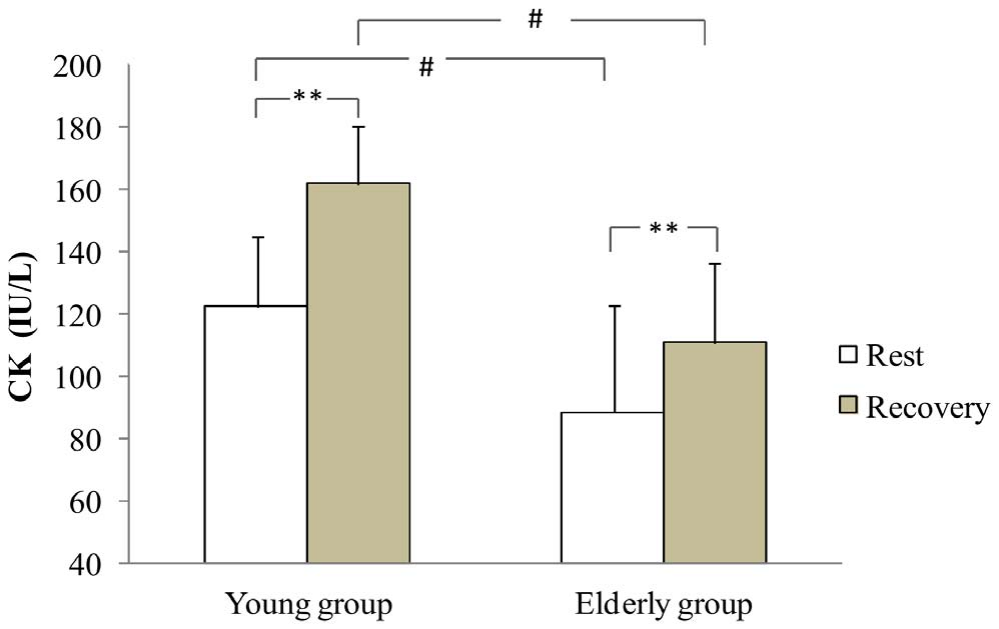
CK at rest and at the recovery in the young and the elderly subjects. **: significant difference between rest and recovery period (p<0.01). #: Significant difference between young and elderly subjects (p<0.05).

**Figure 7:**
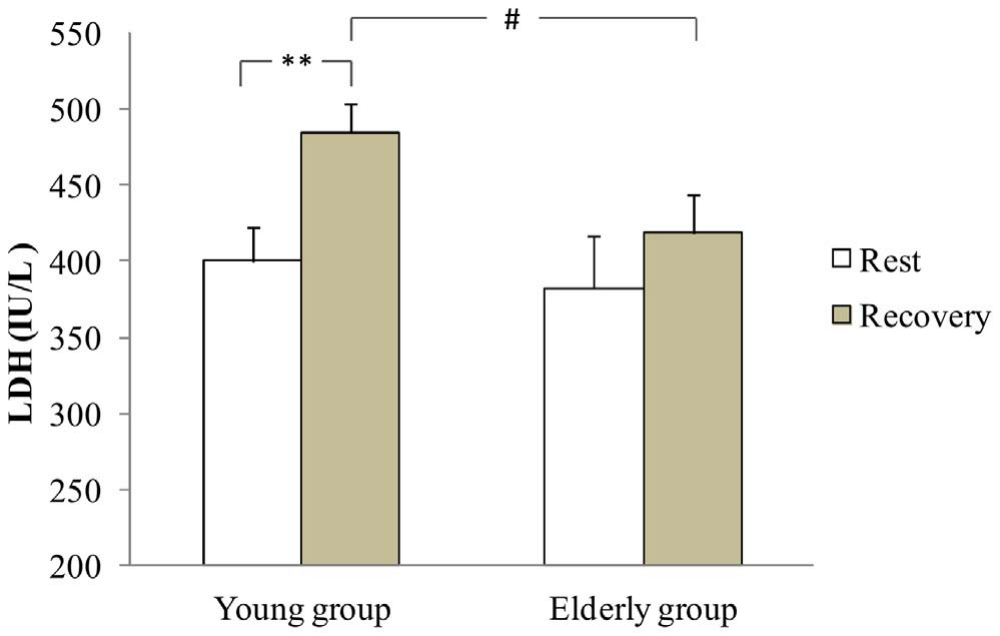
LDH at rest and at the recovery in the young and the elderly subjects. **: Significant difference between rest and recovery period (p<0.01). #: significant difference between young and elderly subjects (p<0.05).

At rest, CK was significantly higher in the young group in comparison with the aged group (p<0.05). During recovery, a significant increase of CK activity was measured in young and older group (+32.2% and +25.1% respectively, p<0.01, Fig. 6). The young group presented a higher CK values than the older group during recovery (p<0.05, Fig. 6). There was no difference in resting LDH in both groups. Moreover, LDH activity increased during recovery only in the young group (+21.2%, p<0.01, Fig. 7). In addition, the young group presented a higher LDH values than the old group during recovery (p<0.05, Fig. 7).

During recovery, SOD was correlated with LDH level (R = 0.67, p<0.01) for the young group. For the elderly group, a significant correlation was shown between LDH and MDA (R = 0.66, p<0.05) and between CK and MDA (R = 0.94, p<0.01). All correlations between oxidative stress markers and biomarkers of muscle damage are presented in the Table 2.

**Table 2:** Correlation between oxidative stress markers and biomarkers of muscle injury at the recovery period.

		SOD		GR		GPX		MDA	
		*R*	*P*	*R*	*P*	*R*	*P*	*R*	*P*
**CK**	Young	0.091	0.752	0.793	0.071	0.074	0.973	0.100	0.731
	Elderly	−0.312	0.294	0.161	0.583	0.040	0.992	0.943	0.000**
**LDH**	Young	0.674	0.009**	0.147	0.615	−0.342	0.232	−0.242	0.941
	Elderly	−0.104	0.747	0.115	0.701	−0.906	0.764	0.664	0.013*

*: Significant correlation at p<0.05.

**: Significant correlation at p<0.01.

## Discussion

The aim of this study was to investigate both resting and exercise-induced oxidative stress markers and biomarkers of muscle damage in elderly and young healthy subjects. The present study results showed a better adaptation of the antioxidant system in the young group through a significant increase in SOD, GPX and GR during the recovery period in comparison with the older group. Moreover, an increase of MDA after the exercise has been shown only in the aged group. On the other hand, biomarkers of muscle injury were higher at rest and during recovery in the young group in comparison with the older adults.

### Physiological measures

Significant difference was observed in VO_2max_ and Pmax between the groups. These results were expected as consequences of the aging phenomena. Shephard et al. [26] summarized that cross sectional research indicated that VO_2max_ decreases fairly steadily in sedentary women and men with average values of 45 ml/kg/min around the age of 20 to about 25 ml/kg/min at the age of 60. The decrease in VO_2max_ with age may be attributed to a decline of maximal heart rate and extraction of oxygen.

### Enzymatic and non enzymatic antioxidants

For non enzymatic antioxidants, similarly to previous findings [10], [27], the present study showed no effects on ascorbic acid and α-Tocopherol levels due to exercise or aging. However, a limitation of the previous studies was that a dietary intake had not been taken into account, and yet it could greatly influence ascorbic acid and α-Tocopherol levels [28], [29]. Also, in view of the results of the literature, it is not possible to conclude if the absence of difference in ascorbic acid and α-Tocopherol levels between groups was due to the dietary intake or to an absence of aging effect. In our study, the absence of significant difference in ascorbic acid and α-Tocopherol intakes between young and older adults leads us to conclude that aging has no effect on these non enzymatic antioxidants.

Regarding enzymatic antioxidants, the present results showed no difference at rest between young and older adults. These data are in accord with findings of Rousseau et al. [30] which reported similar resting antioxidant values (Cu-Zn-SOD, GPX and GR) in young and sedentary older adults. So, aging would not affect antioxidant enzyme activities at rest.

Additionally, the results showed an increase in antioxidant enzymes levels (SOD, GPX, and GR) after exercise only in the young group. This response permits to scavenge the increased production of free radicals assessed from the leakage of electrons in the mitochondrial respiratory chain. The improvement of the leakage of electrons would result from the increase in oxygen consumption during exercise.

However, in the older group, we observed unchanged antioxidants enzymes activities after the exercise. Therefore, two possibilities have to be considered to explain the present findings.

First, the lack of change of antioxidant enzyme reaction in response to acute exercise in the older group may be attributed to an alteration of the redox-sensitive transcription factors with aging [31]. In fact, transcription factors, like NF-Kb and AP-1 are present in the promoter of human enzymatic antioxidants gene and therefore play an important role in antioxidants enzyme activity. Likewise, many studies using animal model have reported an alteration in antioxidants gene transcription factors (NF-Kb and AP-1) with aging [32]. To our knowledge, much less is known about aging effects on antioxidants gene transcription factors in humans.

Secondly, aging could be responsible of a decrease in antioxidants enzymes protein content that could affect antioxidants enzymes activities. In this sense, Sui et al. [33] have reported a decrease in antioxidants enzyme proteins content with aging in animal model. Oh-Ishi et al. [34] have demonstrated that antioxidants enzymes activities seem to be related in part to their protein content. So, this alteration of antioxidants protein content with aging may partly explain the lack of change in antioxidants enzymes activities during recovery in the older group in our study.

### Malondialdehyde

Regarding the effect of exercise on lipid peroxidation, our results show a significant increase in MDA level during recovery only in the aged group. Two possibilities have to be considered to explain the present increase of lipid peroxidation level in the older group following acute exercise.

Firstly, the increase of lipid peroxydation is due to a greater production of free radicals in older subjects. According to Wei et al. [2], a progressive accumulation of somatic mutations in mitochondrial DNA (mtDNA) during the lifetime of an individual leads to a decline in mitochondrial function. ROS produced during normal respiration in a lifetime may produce various modified nucleotides and contribute to the occurrence of somatic mtDNA mutations. In fact, accumulation of mutations and oxidative damage to mtDNA may result in respiratory chain dysfunction, leading to increased production of ROS in mitochondria and induction of further mtDNA mutations [35], [36]. This vicious cycle will gradually diminish the functional capacity of mitochondria and has been proposed to account for an increase in lipid peroxidation in elderly people. Secondly, the degree of fatty acid instauration affects lipid peroxidation level. Unsaturated fatty acids are the cellular macromolecules most sensitive to ROS damage, owing to the presence of highly unstable electrons near their double bonds, and their sensitivity to lipid peroxidation exponentially increases as a function of the number of double bonds per molecule [37]. Thus, a high level of fatty acid instauration would increase lipid peroxidation level. In this sense, Schäfer et al. [38] have noted a higher unsaturated fatty acid level in elderly subjects in comparison with young subjects. In this context, Balkan et al. [39] have found a positive correlation between polyunsaturated fatty acid level and lipid peroxidation marker (TBARS) in elderly subjects, which may explain the link between higher unsaturated fatty acid level and increased lipid peroxidation with aging.

### Biomarkers of muscle damage

At rest, the results of the present study show that CK level was higher in the young group in comparison to the aged group with no significant difference in LDH level between both groups. These data are in accord with the work of Lavender et al. [9] which reported a higher level of CK in young subjects in comparison with older subjects at baseline. These results may be a consequence of the smaller muscle mass of the elderly subjects.

Regarding the responses following the incremental exercise on markers of muscle damage, the present results show a significant increase of CK activity during recovery in both groups associated with a significant lower level in the older group. Also, LDH level has increased only in the young group during the recovery period. These results demonstrated a higher muscle damage response in the young group to acute exercise. Difference in response between the older and the young group in our study may be explained by a decrease in muscle content in type II fibers linked with aging [9]. It has been documented that type II fibers are more susceptible to muscle damage than type I fibers [40]. Lower levels of muscle damage response in the older group may be explained by their lower muscle content of type fibers II. The results of the present study may indicate that older adults are not more susceptible to exercise-induced muscle injury than young people.

On the other hand, during recovery, changes in biomarkers of muscle injury in both groups were correlated with an alteration in oxidative stress markers. We have noticed a significant correlation between biomarkers of muscle injury (CK and LDH) and MDA in the older adults group. To our knowledge, no study has investigated correlation between lipid peroxidation markers and biomarkers of muscle injury after acute exercise in older adults. Elevation of plasmatic CK and LDH levels are characteristic responses to strenuous exercise and are often used as indicators of muscle damage [41]. MDA is one of the major peroxidation products derived from polyunsaturated fatty acids so that the measurement of this marker has been used as an index of structural oxidative injury of the cell membrane. The increase in MDA in the older group during recovery suggests increased lipid peroxidation initiated by free radical reactions and could be a direct reflection of an oxidative injury of the skeletal muscle [42]. The correlation between MDA and biomarkers of muscle injury during recovery might result from an increase in membrane permeability due to lipid peroxidation in the older group. Nonetheless, absence of correlation between makers of muscle injury and lipid peroxidation in the young group during recovery may be attributed to their better antioxidants defense which may reduce lipid peroxidation by scavenging free radicals produced during exercise. However, this rise in antioxidants activity in the young group could, partly participate in the muscle injury processes during recovery and explain the positive correlation between SOD and CK in our study. In fact, SOD catalyzes the conversion of superoxide radicals (O_2_^−^) into H_2_O_2_. H_2_O_2_ is considered as a reactive species because of its toxicity and capacity to cause reactive species formation. In leukocytes, myeloperoxidase transforms H_2_O_2_ in hypochlorous acid (HOCl^−^), one of the strongest physiological oxidants [43]. These reactants (HOCl^−^), if they stay unchecked, can also destroy adjacent healthy muscle tissue and therefore increase muscle damage markers level such as CK and LDH.

### Methodological limitations

To the best of our knowledge, this is the first study to explore aging effects on both resting and exercise-induced oxidative stress markers and biomarkers of muscle damage. However, some limitations inherent to the experimental protocol of this study warrant mention. First, it is possible that oxidative stress may have occurred in tissues aside from blood, such as skeletal muscle, which may be the ideal tissue when studying exercise stress. Of course, biopsies are required for obtaining samples for analyses, which is likely the reason why so few human investigations include the analysis of oxidative stress biomarkers in skeletal muscle [24]. As reported previously using an animal model, changes in oxidative stress observed during the postexercise period may not be the same when comparing blood and skeletal muscle [44]. Secondly, we only measured selected biomarkers of oxidative stress and muscle injury and did not include an exhaustive list of potential markers (F2-isoprostanes, 8-OHdG, myoglobine.). Although our array of biomarkers is well able to characterize the oxidative status and muscle damage during the postexercise period, the inclusion of additional biomarkers may have strengthened the results of this study.

## Conclusion

The present study demonstrates that young people have a higher endogenous antioxidant capacity as compared with older subjects following an exhaustive exercise. This can be explained by a decrease in antioxidants efficiency with aging. Moreover, this study shows that aging is associated with increased lipid peroxidation which may be due to a rise in ROS production associated with a decrease in antioxidant defense. Finally, these results tend to demonstrate that older adults are not more susceptible to muscle damage induced by acute exercise in comparison with young subjects.

## References

[1] Wang CH, Wu SB, Wu YT, Wei YH (2013) Oxidative stress response elicited by mitochondrial dysfunction: implication in the pathophysiology of aging. Exp Biol Med (Maywood) 238: 450–60. 23856898 10.1177/1535370213493069

[2] Wei YH, Lee HC (2002) Oxidative stress, mitochondrial DNA mutation, and impairment of antioxidant enzymes in aging. Exp Biol Med 227: 671–82. 10.1177/153537020222700901 12324649

[3] Mutlu-Türkoğlu U, Ilhan E, Oztezcan S, Kuru A, Aykaç-Toker G, et al (2003) Age related increases in plasma malondialdehyde and protein carbonyl levels and lymphocyte DNA damage in elderly subjects. Clin Biochem 36: 397–400. 12849873 10.1016/s0009-9120(03)00035-3

[4] Kasapoglu M, Ozben T (2001) Alterations of antioxidant enzymes and oxidative stress markers in aging. Exp Gerontol 36: 209–20. 11226737 10.1016/s0531-5565(00)00198-4

[5] Gianni P, Jan KJ, Douglas MJ, Stuart PM, Tarnopolsky MA (2004) Oxidative stress and the mitochondrial theory of aging in human skeletal muscle. Exp Gerontol 39: 1391–400. 15489062 10.1016/j.exger.2004.06.002

[6] Karaouzene N, Merzouk H, Aribi M, Merzouk SA, Berrouiguet AY, et al (2011) Effects of the association of aging and obesity on lipids, lipoproteins and oxidative stress biomarkers: a comparison of older with young men. Nutr Metab Cardiovasc Dis 21: 792–9. 20554180 10.1016/j.numecd.2010.02.007

[7] Meng SJ, Yu LJ (2010) Oxidative stress, molecular inflammation and sarcopenia. Int J Mol Sci 12: 1509–26. 10.3390/ijms11041509 PMC2871128 20480032

[8] Figueiredo PA, Powers SK, Ferreira RM, Appell HJ, Duarte JA (2009) Aging impairs skeletal muscle mitochondrial bioenergetic function. J Gerontol A Biol Sci Med Sci 64: 21–33. 19196905 10.1093/gerona/gln048 PMC2691197

[9] Lavender AP, Nosaka K (2006) Comparison between old and young men for changes in makers of muscle damage following voluntary eccentric exercise of the elbow flexors. Appl Physiol Nutr Metab 31: 218–25. 16770348 10.1139/h05-028

[10] Sacheck J, Milbury PE, Cannon JG, Roubenoff R, Blumberg JB (2003) Effect of vitamin E and eccentric exercice on selected biomarkers of oxidative stress on young and elderly men. Free Radic Biol Med 34: 1575–1588. 12788477 10.1016/s0891-5849(03)00187-4

[11] Powers SK, Jackson MJ (2008) Exercise-induced oxidative stress: cellular mechanisms and impact on muscle force production. Physiol Rev 88: 1243–1276. 18923182 10.1152/physrev.00031.2007 PMC2909187

[12] Di Massimo C, Taglieri G, Penco M, Tozzi-Ciancarelli MG (1999) Influence of aging and exercise-induced stress on human platelet function. Clin Hemorheol Microcirc 20: 105–110. 10416812

[13] Toft AD, Jensen LB, Bruunsgaard H, Ibfelt T, Halkjaer-Kristensen J, et al (2002) Cytokine response to eccentric exercise in young and elderly humans. Am J Physiol Cell Physiol 283: 289–95. 10.1152/ajpcell.00583.2001 12055098

[14] Bloomer RJ, Goldfarb AH (2004) Anaerobic exercise and oxidative stress: A review. Can J Appl Physiol 29: 245–63. 15199226 10.1139/h04-017

[15] Frankiewicz-Jóźko A, Faff J, Sieradzan-Gabelska B (1996) Changes in concentrations of tissue free radical marker and serum creatine kinase during the post-exercise period in rats. Eur J Appl Physiol Occup Physiol 74: 470–4. 8954295 10.1007/BF02337728

[16] Williams C, Kronfeld D, Hess T, Saker K, Waldron J, et al (2004) Antioxidant supplementation and subsequent oxidative stress of horses during an 80-km endurance race. J Anim Sci 82: 588–594. 14974559 10.2527/2004.822588x

[17] Ji LL, Dillon D, Wu E (1990) Alteration of antioxidant enzymes with aging in rat skeletal muscle and liver. Am J Physiol 258: 918–23. 10.1152/ajpregu.1990.258.4.R918 2331035

[18] Bejma J, Ramires P, Ji LL (2000) Free radical generation and oxidative stress with ageing and exercise: differential effects in the myocardium and liver. Acta Physiol Scand 169: 343–51. 10951126 10.1046/j.1365-201x.2000.00745.x

[19] Fletcher GF, Balady GJ, Amsterdam EA, Chaitman B, Eckel R, et al (2001) Exercise standards for testing and training: a statement for healthcare professionals from the American Heart Association. Circulation 104: 1694–1740. 11581152 10.1161/hc3901.095960

[20] Prasertsri P, Roengrit T, Kanpetta Y, Tong-Un T, Muchimapura S, et al (2013) Cashew apple juice supplementation enhanced fat utilization during high-intensity exercise in trained anduntrained men. J Int Soc Sports Nutr 1: 10–13. 10.1186/1550-2783-10-13 PMC3610290 23497120

[21] Viña J, Gomez-Cabrera MC, Lloret A, Marquez R, Miñana JB, et al (2000) Free radicals in exhaustive physical exercise: mechanism of production, and protection by antioxidants. IUBMB Life 50: 271–7. 11327321 10.1080/713803729

[22] Tanaka H, Monahan KD, Seals DR (2001) Age predicted maximal heart rate revisited. J Am Coll Cardiol 37: 153–6. 11153730 10.1016/s0735-1097(00)01054-8

[23] Hammouda O, Chtourou H, Chahed H, Ferchichi S, Kallel C, et al (2011) Diurnal variations of plasma homocysteine, total antioxidant status,and biological markers of muscle injury during repeated sprint: effect on performance and muscle fatigue: a pilot study. Chronobiol Int 28: 958–67. 22080741 10.3109/07420528.2011.613683

[24] Farney TM, McCarthy CG, Canale RE, Schilling BK, Whitehead PN, et al (2012) Absence of blood oxidative stress in trained men after strenuous exercise. Med Sci Sports Exerc 44: 1855–63. 22525774 10.1249/MSS.0b013e3182592575

[25] El Abed K, Rebai H, Bloomer RJ, Trabelsi K, Masmoudi L, et al (2011) Antioxidant status and oxidative stress at rest and in response to acute exercise in judokas and sedentary men. J Strength Cond Res 25: 2400–2409. 21869626 10.1519/JSC.0b013e3181fc5c35

[26] Shephard RJ (2009) Maximal oxygen intake and independence in old age. Br J Sports Med 43: 342–6. 18403414 10.1136/bjsm.2007.044800

[27] Meydani M, Evans WJ, Handelman G, Biddle L, Fielding RA, et al (1993) Protective effect of vitamin E on exercise-induced oxidative damage in young and older adults. Am J Physiol 264: 992–8. 10.1152/ajpregu.1993.264.5.R992 8498608

[28] Fang YZ, Yang S, Wu G (2002) Free radicals, antioxidants, and nutrition. Nutrition 18: 872–9. 12361782 10.1016/s0899-9007(02)00916-4

[29] Schmuck A, Ravel A, Coudray C, Alary J, Franco A, et al (1996) Antioxidant vitamins in hospitalized elderly patients: analysed dietary intakes and biochemical status. Eur J Clin Nutr 50: 473–8. 8862485

[30] Rousseau AS, Margaritis I, Arnaud J, Faure H, Roussel AM (2006) Physical activity alters antioxidant status in exercising elderly subjects. J Nutr Biochem 17: 463–70. 16443361 10.1016/j.jnutbio.2005.10.001

[31] Lavrovsky Y, Chatterjee B, Clark RA, Roy AK (2000) Role of redox-regulated transcription factors in inflammation, aging and age-related diseases. Exp Gerontol 35: 521–32. 10978675 10.1016/s0531-5565(00)00118-2

[32] Bar-Shai M, Carmeli E, Ljubuncic P, Reznick AZ (2008) Exercise and immobilization in aging animals: the involvement of oxidative stress and NF-kappaB activation. Free Radic Biol Med 15: 202–14. 10.1016/j.freeradbiomed.2007.03.019 18191756

[33] Siu PM, Pistilli EE, Alway SE (2008) Age dependent increase in oxidative stress in gastrocnemius muscle with unloading. J Appl Physiol 105: 1695–705. 18801960 10.1152/japplphysiol.90800.2008 PMC2612472

[34] Oh-ishi S, Kizaki T, Nagasawa J, Izawa T, Komabayashi T, et al (1997) Effects of endurance training on superoxide dismutase activity, content and mRNA expression in rat muscle. Clin Exp Pharmacol Physiol 24: 326–32. 9143782 10.1111/j.1440-1681.1997.tb01196.x

[35] Hou JH, Wei YH (1996) The unusual structures of the hot-regions flanking large-scale deletions in human mitochondrial DNA. Biochem J 318: 1065–1070. 8836157 10.1042/bj3181065 PMC1217724

[36] Lee HC, Wei YH (2001) Mitochondrial alterations, cellular response to oxidative stress and defective degradation of proteins in aging. Biogerontology 2: 231–244. 11868898 10.1023/a:1013270512172

[37] Barja G (2004) Free radicals and aging. Trends Neurosci 27: 595–600. 15374670 10.1016/j.tins.2004.07.005

[38] Schäfer L, Overvad K, Thorling EB, Velander G (1989) Adipose tissue levels of fatty acids and tocopherol in young and old women. Ann Nutr Metab 33: 315–22. 2619256 10.1159/000177552

[39] Balkan J, Kanbağli O, Mehmetçik G, Mutlu-Türkoğlu U, Aykaç-Toker G (2002) Increased lipid peroxidation in serum and low-density lipoproteins associated with aging in humans. Int J Vitam Nutr Res 72: 315–20. 12463107 10.1024/0300-9831.72.5.315

[40] Monemi M, Eriksson PO, Eriksson A, Thornell LE (1998) Adverse changes in fibre type composition of the human masseter versus biceps brachii muscle during aging. J Neurol Sci 154: 35–48. 9543320 10.1016/s0022-510x(97)00208-6

[41] Kanter MM, Lesmes GR, Kaminsky LA, La Ham-Saeger J, Nequin ND (1988) Serum creatine kinase and lactate dehydrogenase changes following an eighty kilometer race. Relationship to lipid peroxidation. Eur J Appl Physiol Occup Physiol 57: 60–3. 3342795 10.1007/BF00691239

[42] Fantini AG, Yoshioka T (1993) Desferroxamine prevents lipid peroxidation and attenuates reoxygenation injury in postischemic skeletal muscle. Am J Physiol 264: 1953–1959. 10.1152/ajpheart.1993.264.6.H1953 8322925

[43] Nikolaidis MG, Paschalis V, Giakas G, Fatouros IG, Koutedakis Y, et al (2007) Decreased blood oxidative stress after repeated muscle-damaging exercise. Med Sci Sports Exerc 39: 1080–9. 17596775 10.1249/mss.0b013e31804ca10c

[44] You T, Goldfarb AH, Bloomer RJ, Nguyen L, Sha X, et al (2005) Oxidative stress response in normal and antioxidant supplemented rats to a downhill run: changes in blood and skeletal muscles. Can J Appl Physiol 30: 677–89. 16485519 10.1139/h05-148

